# Intraoperative Cytokines and Postcraniotomy Infection in Benign Brain Tumors: An Exploratory Prospective Study

**DOI:** 10.3390/jcm15135119

**Published:** 2026-07-01

**Authors:** Mingfei Wang, Siyao Li, Mengjuan Chai, Xin Pi

**Affiliations:** 1 Department of Neurology, The First Affiliated Hospital of Harbin Medical University, Harbin 150001, China; 813917@hrbmu.edu.cn; 2 Department of Anesthesiology, The First Affiliated Hospital of Harbin Medical University, Harbin 150001, China; 2024021093@hrbmu.edu.cn (S.L.); 2025020979@hrbmu.edu.cn (M.C.)

**Keywords:** intracranial infection, cerebrospinal fluid, intraoperative cytokines, exploratory study

## Abstract

**Objective:** Intracranial infection is a severe complication that can occur following neurosurgery, and early diagnosis is crucial for improving patient prognosis. In this study, we aimed to investigate, from an exploratory perspective, whether the immune microenvironment of intraoperative cerebrospinal fluid (CSF) is associated with postoperative intracranial infection (PII) in patients undergoing craniotomy for benign brain tumors. **Methods:** A total of 134 patients undergoing neurosurgery for benign brain tumors were included and categorized into an infection group (*n* = 18) and a non-infection group (*n* = 116). CSF samples were collected aseptically immediately after dural opening during surgery. The concentrations of 16 cytokines, including monocyte chemoattractant protein-1 (MCP-1); macrophage inflammatory protein-1α (MIP-1α) and MIP-1β; interleukin (IL)-1α, IL-1β, IL-4, IL-6, IL-8, IL-10, IL-12, IL-13, and IL-17; interferon (IFN)-α and IFN-γ; tumor necrosis factor-α (TNF-α); and granulocyte colony-stimulating factor (G-CSF), were quantified using Cytometric Bead Array (CBA) technology. An independent samples *t*-test was used for normally distributed data, while the Mann–Whitney U test was applied for non-normally distributed data. Group comparisons were performed using independent-samples *t*-tests or Mann–Whitney U tests for continuous variables and χ^2^ tests or Fisher’s exact tests for categorical variables. The Benjamini–Hochberg false discovery rate (FDR) correction was applied to all 16 cytokines to control for multiple testing. Receiver operating characteristic (ROC) curve analysis was performed to assess discriminatory capacity. Statistical significance was defined as *p* < 0.05. **Results:** PII developed in 18 of 134 patients (13.4%). Age (47.78 vs. 54.86, *p* = 0.028) and operative duration (390 vs. 244 min, *p* = 0.005) showed differences in unadjusted analyses. In the unadjusted comparisons, MCP-1 and IL-4 levels were found to be significantly lower in the infection group (MCP-1: 57.78 vs. 116.03 pg/mL, *p* = 0.003; IL-4: 24.38 vs. 28.18 pg/mL, *p* = 0.032). No cytokine remained significant after FDR correction. The ROC analysis showed that age and IL-4 demonstrated mild discriminatory performance, with AUC values of 0.665 (95% CI 0.526–0.803, *p* = 0.025) and 0.657 (95% CI 0.540–0.774, *p* = 0.032), while MCP-1 and operative duration demonstrated modest discriminatory performance, with AUC values of 0.716 (95% CI 0.595–0.838, *p* = 0.003) and 0.708 (95% CI 0.578–0.838, *p* = 0.002). **Conclusions:** In this study, single-point intraoperative CSF cytokines were not significantly associated with PII after stringent correction for multiple testing, and did not provide a validated clinical prediction tool. The unadjusted and direction-corrected findings for MCP-1 and IL-4 remain exploratory and require validation.

## 1. Introduction

Postoperative intracranial infections (PIIs), such as bacterial meningitis and ventriculitis, remain a formidable clinical challenge following neurosurgery. Although the incidence of such infections is relatively low, their consequences are devastating [1]. PII often leads to significantly prolonged hospitalization, exacerbated neurological deficits, increased healthcare costs, and markedly increased morbidity and mortality [2]. However, early clinical diagnosis of PII remains challenging. Typical symptoms, such as fever, headache, and neck stiffness, are often confounded by postoperative physiological reactions, including postoperative absorption fever and surgical traumatic pain [3,4]. Currently, the gold standard for diagnosis remains CSF culture. Nevertheless, its clinical sensitivity has been reduced due to early empirical antibiotic use, and performing CSF culturing is time-consuming [5]. Moreover, routine CSF biochemical parameters, such as increased white blood cell counts, increased protein levels, and decreased glucose concentrations, lack specificity in the early postoperative period [6].

Established risk factors, such as prolonged surgery duration, CSF leak, diabetes mellitus, and reoperation, can partially explain individual susceptibility, suggesting that host immunological factors may play a critical role [7]. As CSF bathes the entire CNS compartment, its molecular and cellular composition serves as a local immune microenvironment, reflecting ongoing neuroinflammatory or immunomodulatory processes. Primary brain tumors can actively sculpt an immunosuppressive niche to subvert host immunity. Previous reports have demonstrated that glioblastoma can secrete anti-inflammatory cytokines and recruit regulatory T cells, myeloid-derived suppressor cells, and M2-polarized macrophages [8,9]. However, the relationship between the CSF immune microenvironment and the subsequent development of intracranial infection has not been systematically investigated in this patient population.

Inflammatory cytokines are central mediators of the host response to pathogen invasion and play a pivotal role in the pathophysiology of infectious diseases. In bacterial central nervous system infections, pathogens and their components activate microglia, astrocytes, and infiltrating leukocytes, leading to the cascade release of pro-inflammatory cytokines (e.g., TNF-α, IL-1β, IL-6, IL-8) and chemokines (e.g., MCP-1, MIP-1α) [10,11,12]. Concurrently, the feedback upregulation of anti-inflammatory cytokines (such as IL-10, IL-4, and IL-13) is essential for modulating inflammation and preventing excessive tissue injury [10]. However, the relationship between baseline levels of cytokines during surgery and the occurrence of postoperative intracranial infections has not been reported.

In this study, we enrolled a cohort of patients undergoing neurosurgery for treating benign brain tumors. CSF specimens were collected uniformly during surgery (immediately after dural opening) to reflect the local immune microenvironment. We simultaneously quantified 16 key cytokines covering distinct functional categories, including major chemokines (MIP-1α, MIP-1β, MCP-1), classical pro-inflammatory factors (TNF-α, IL-1, IL-6, IL-8, IL-12, IL-17, IFN-γ), anti-inflammatory/Th2-type factors (IL-4, IL-10, IL-13), and immunomodulatory factors (IFN-α, G-CSF). We hypothesized that patients who develop PII would exhibit detectable and characteristic dysregulation of inflammatory cytokines in their intraoperative CSF. This study was therefore designed to explore the association between intraoperative CSF immune cytokine networks and PII following the resection of benign brain tumors.

## 2. Materials and Methods

Study Design and Ethics: This study was a single-center, prospective observational cohort study. Individuals who underwent surgical resection for benign brain tumors in the Department of Neurosurgery at the First Affiliated Hospital of Harbin Medical University between 1 September 2024 and 1 January 2026 were recruited as the study subjects. All patients underwent craniotomy for tumor resection. For all patients, cefazolin antibiotics were intravenously administered half an hour before surgery. When the operative duration exceeded 4 h, an additional dose of antibiotic was given. No patients exhibited ventricular opening, CSF drainage, CSF leakage, ICU admission, or reoperation. The study protocol was approved by the Review Committee of the First Affiliated Hospital of Harbin Medical University (Approval No.: 2024220). All included patients or their legal representatives provided informed consent.

The inclusion criteria were as follows: (1) age ≥ 18 years; (2) planned elective craniotomy for tumor resection due to the imaging and/or pathological confirmation of benign brain tumors (meningioma or schwannoma).

The exclusion criteria were as follows: (1) presence of active systemic or intracranial infection prior to surgery; (2) use of systemic antibiotics or glucocorticoids within 1 week before surgery; (3) diagnosis of autoimmune diseases, hematologic malignancies, or other severe immune dysfunction disorders; (4) concurrent malignant tumors or life expectancy less than 3 months; (5) missing detection data.

Data collection: We collected baseline patient data, including gender, age, hypertension status, diabetes status, operative duration, surgical blood loss volume, tumor type and surgical site.

Specimen collection and processing: During the surgical procedure, the neurosurgeon incised the dura mater and confirmed cerebrospinal fluid (CSF) outflow, before collecting 2–3 mL of CSF using aseptic techniques. The specimen was immediately placed on ice and delivered to the laboratory within 30 min. In a biosafety cabinet, the CSF was centrifuged at 3000 rpm for 10 min (4 °C) to separate the supernatant. The supernatant was aliquoted into sterile cryovials and stored in an ultra-low-temperature freezer at −80 °C until batch testing. All assays were performed strictly according to the instructions for using the cytokine kit (Aptplex™ Human Neuroinflammation 16-Plex Panel, Elabscience, Wuhan, China). The experiment incorporated blank controls, standard references, and duplicate parallel controls. All samples were analyzed within the same batch to effectively reduce batch effects. The minimum detectable doses of human MIP-1β, MIP-1α, MCP-1, IL-17, IL-13, IL-12, IL-10, IL-8, IL-6, IL-1β, IL-1α and G-CSF are typically less than 0.3255 pg/mL, 0.3255 pg/mL, 0.3255 pg/mL, 0.3255 pg/mL, 0.3255 pg/mL, 0.0651 pg/mL, 0.1628 pg/mL, 0.3255 pg/mL, 0.1628 pg/mL, 0.3255 pg/mL, 0.1628 pg/mL, 0.3255 pg/mL, and 0.3255 pg/mL. The minimum detectable doses of human TNF-α, IL-4, and IFN-α are typically less than 0.327 pg/mL, 0.327 pg/mL, and 0.163 pg/mL. For low-abundance cytokines that were below the instrument’s detection limit, standardized values were assigned according to the kit’s minimum detection limit to reduce missing values. Three cytokines were detected below the lower limit values, including MCP-1b, IL-17, and IL-1α, with proportions of 0.04%, 0.02%, and 0.01%, respectively.

Diagnosis and classification of intracranial infection: Postoperatively, infectious disease and neurosurgery physicians who were unaware of the study results referred to the diagnostic criteria outlined in the “China Expert Consensus on Diagnosis and Treatment of Central Nervous System Infections in Neurosurgery 2021” [13]. They combined these criteria with clinical manifestations, laboratory tests, and microbiological results to jointly evaluate and diagnose intracranial infection. A diagnosis of intracranial infection required meeting at least one of the following criteria: (1) pathogenic microorganisms were cultured from cerebrospinal fluid; (2) clinical symptoms such as fever (>38 °C), headache, neck stiffness, and altered consciousness status were present, accompanied by a significantly elevated cerebrospinal fluid white blood cell count (typically >100 × 10^6^/L, predominantly neutrophils), decreased glucose levels (<2.2 mmol/L or below 40% of the corresponding blood glucose level), and increased protein levels (>0.45 g/L), regardless of culture positivity. Patients were divided into the intracranial infection group and non-infection group based on whether intracranial infection was confirmed.

## 3. Statistical Analysis

Data analysis was performed using SPSS statistical software (version 26.0, IBM Corp., Armonk, NY, USA). Data conforming to the normal distribution were expressed as mean ± standard deviation (Mean ± SD), with intergroup comparisons conducted via an independent samples *t*-test. Data not conforming to the normal distribution were expressed as the median and interquartile range [M(IQR)], and intergroup comparisons were performed using the Mann–Whitney U test. Categorical data were presented as frequencies (percentages), with intergroup comparisons analyzed using the chi-square test or Fisher’s exact test. Univariate logistic regression and multiple testing correction were employed to identify potential indicators associated with postoperative infection, and the Benjamini–Hochberg false discovery rate (FDR) correction was applied subsequently. The area under the curve (AUC), optimal cut-off value, sensitivity, specificity, and Youden index were determined according to the ROC curve. A *p*-value < 0.05 was considered statistically significant.

## 4. Results

1. Patient baseline characteristics

**Figure 1:**
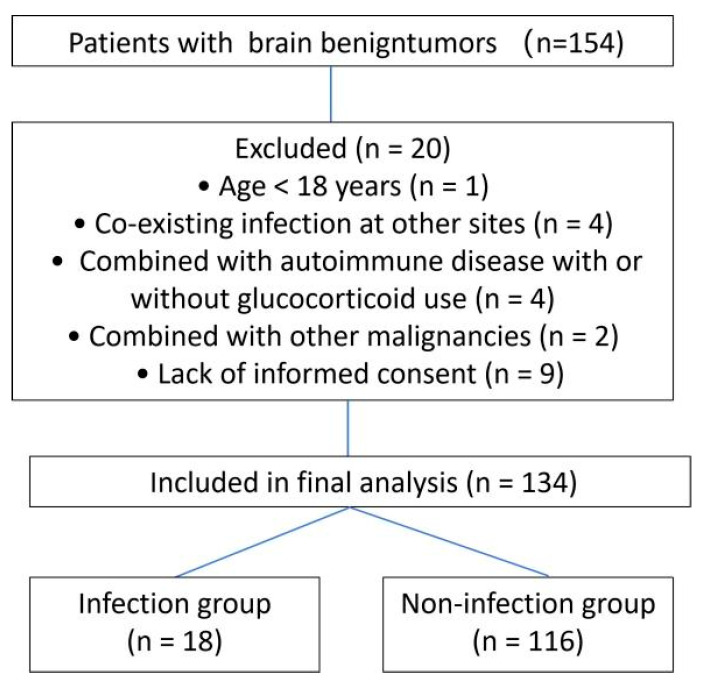
A flow diagram of participant enrollment.

A total of 154 patients were initially screened, and 134 patients met the inclusion and exclusion criteria and completed this study (see Figure 1 for more details). Among these, 18 patients (incidence rate of 13.4%) were diagnosed with postoperative intracranial infection, while 116 remained non-infected. The clinical data of patients with PII are presented in Supplementary Materials Table S1. The mean time to diagnosis of intracranial infection in the infection group was 4.7 ± 1.7 days. Within the infection group, CSF cultures were positive in four cases, with pathogens identified as Staphylococcus aureus (*n* = 3) and Klebsiella pneumoniae (*n* = 1). Statistically significant differences were observed between the infection and non-infection groups in terms of age (47.78 vs. 54.86, *p* = 0.028) and operative duration (390 vs. 244 min, *p* = 0.005). In contrast, no significant differences were found regarding sex, hypertension, diabetes, tumor type, surgical site, or intraoperative blood loss (all *p* > 0.05; Table 1).

2. Comparison of CSF cytokine levels

The data revealed no statistically significant differences between the intracranial infection and non-infection groups regarding the concentrations of MIP-1β, MIP-1α, IL-17, IL-13, IL-12, IL-10, IL-8, IL-6, IL-1β, IL-1α, IFN-γ, G-CSF, TNF-α, and IFN-α (all *p* > 0.05). However, significant differences were observed for MCP-1 and IL-4. Both MCP-1 and IL-4 levels were significantly lower in the infection group compared to the non-infection group (MCP-1: 57.78 vs. 116.03 pg/mL, *p* = 0.003; IL-4: 24.38 vs. 28.18 pg/mL, *p* = 0.032). However, MCP-1 and IL-4 did not retain statistical significance via the FDR method (Table 1).

3. Univariate analysis of variables and intracranial infection after surgery

ORs are reported in clinically interpretable units. Multiple variables exhibited significant *p*-values in the initial analysis, including age (OR: 0.67, 95% CI: 0.46–0.97, *p* = 0.033), operative duration (OR: 1.19, 95% CI: 1.05–1.34, *p* = 0.005), MCP-1 (OR: 0.92, 95% CI: 0.86–1.00, *p* = 0.043), and IL-4 (OR: 0.36, 95% CI: 0.14–0.96, *p* = 0.041). However, MCP-1 and IL-4 did not retain statistical significance subsequent to correction for multiple testing via the FDR method (both FDR-corrected *p* > 0.05). The clinical variables, including age, gender, operative duration, blood loss, tumor type, surgical site, hypertension, and diabetes were based on well-defined clinical a priori assumptions with specific expected effect directions for each variable. Therefore, no multiple comparison correction was performed for these variables. The results are shown in Table 2. More extensive and well-powered studies are necessary to clarify whether MCP-1 and IL-4 variables possess clinical relevance.

4. ROC curve analysis of age, MCP-1, IL-4, and operative duration for postoperative intracranial infection

ROC curve analysis was conducted to assess the discriminatory ability of age, MCP-1, IL-4, and operative duration with respect to postoperative intracranial infection. Based on the biological effects of various inflammatory factors and clinical indicators, ROC curves were predefined to determine the relationship: lower values for age, MCP-1, and IL-4 were associated with higher infection risk, whereas higher values for operative duration were associated with higher infection risk. Age and IL-4 demonstrated mild discriminatory performance, with AUC values of 0.665 (95% CI 0.526–0.803, *p* = 0.025) and 0.657 (95% CI 0.540–0.774, *p* = 0.032), while MCP-1 and operative duration demonstrated modest discriminatory performance, with AUC values of 0.716 (95% CI 0.595–0.838, *p* = 0.003) and 0.708 (95% CI 0.578–0.838, *p* = 0.002). The optimal cut-off values of MCP-1 and operative duration were 80.82 pg/mL and 389.5 min, with sensitivities of 77.8% and 55.6% and specificities of 67.2% and 86.2%, respectively. The results are shown in Table 3. However, given the limited sample size and lack of significance after multiple-testing correction, these findings should be regarded as exploratory. Therefore, internal cross-validation or external cohort validation was not performed. The analysis was limited to exploring the ability of indicators to differentiate between the intracranial infection groups, and no inference of a clinical prediction model was intended.

## 5. Discussion

In this exploratory prospective investigation, we explored whether the intraoperative CSF cytokine microenvironment, sampled immediately after dural opening, is associated with the subsequent occurrence of PII in patients undergoing craniotomy for benign brain tumors. In this cohort, the PII incidence reached 13.4%, within the normal range for postoperative infections following craniocerebral tumor surgery [14]. Age and operative duration showed a statistically significant association with intracranial infection. In unadjusted comparisons, the intraoperative concentrations of MCP-1 and IL-4 were significantly lower in the infection group. However, after applying the Benjamini–Hochberg FDR correction for multiple testing, neither of these two cytokines retained a statistically significant association with PII. Age and IL-4 demonstrated mild discriminatory performance, while MCP-1 and operative duration demonstrated modest discriminatory performance under the ROC curve. Yet, the significance of MCP-1 and IL-4 was also negated by FDR correction.

MCP-1, an important inflammatory mediator secreted by various human cells, can chemotactically attract monocytes and promote the initiation and progression of inflammatory responses [15], suggesting that MCP-1 plays a critical role in the occurrence and progression of intracranial infections following intracranial tumor resection [16,17]. IL-4 exerts a dual “anti-inflammatory and protective” effect in intracranial infections by regulating immune cell polarization, inhibiting pro-inflammatory pathways [18,19]. Its expression levels are closely associated with infection type, disease severity, and prognosis. In the tumor microenvironment, IL-4 sourced from Th2 cells and innate lymphoid cells can polarize macrophages toward an M2-like phenotype, contributing to the creation of an immunosuppressive niche [20]. However, the relationship between intraoperative CSF MCP-1 and IL-4 levels and PII has not been reported. The unadjusted comparison of intraoperative CSF MCP-1 and IL-4 revealed a statistically significant reduction in the infection group. One hypothetical explanation is that a lower baseline MCP-1 and IL-4 could signify a local innate immune deficit, potentially shaped by the tumor’s immunosuppressive secretome. However, both cytokines failed to withstand multiple testing adjustment. Taken together, these observations suggest that the exploratory cytokine signals observed here are tentative.

The current findings diverge from the hypothesis that the CSF immune microenvironment, sculpted by a benign brain tumor, is relevant to infectious vulnerability. Several methodological and biological considerations may account for this result. First, the timing of specimen collection may be a pivotal factor. In this study, CSF was collected intraoperatively, typically preceding the onset of clinical infection symptoms. It does not reflect the dynamic immune reserve and the capacity to mount an acute cytokine burst upon bacterial challenge. Second, the analytical strategy employed applied stringent FDR correction to counteract the high risk of false discovery inherent in multiplexed cytokine studies. Third, the diagnosis of PII itself introduced further heterogeneity: only 4 of 18 infections showed positive cultures, with the remainder defined by Chinese Expert Consensus Guidelines. The low culture positive rate may be attributed to prior antibiotic use before surgery or low bacterial burden. Future studies would benefit from conducting preplanned sensitivity analyses restricted to culture-confirmed cases or incorporating metagenomic next-generation sequencing (mNGS) to refine pathogen detection [21].

In this study, we observed that younger age may be associated with postoperative intracranial infection. ROC analysis suggested limited discriminative ability. This observation contrasts with traditional assumptions that advanced age increases infection risk. Possible explanations include differing surgical indications, stronger inflammatory responses potentially disrupting tissue barriers in younger patients, or disparities in perioperative management intensity. However, given the small sample size and single-center design, these findings should be interpreted cautiously and warrant validation in larger cohorts. Age alone is insufficient for risk stratification and must be considered alongside established factors.

Prolonged surgery is a well-replicated risk factor for neurosurgical site infection, attributed to greater cumulative anesthesia exposure, increased surgical stress and systemic inflammation, higher rates of postoperative cerebral edema, and a greater burden of medical complications [22,23,24]. In this study, all patients received a single intravenous dose of prophylactic antibiotics half an hour before surgery, with intraoperative re-dosing performed if the operation lasted more than 4 h. Nevertheless, it remains to be determined whether extended prophylactic regimens provide additional protective advantages in high-risk, long-duration craniotomies. Future prospective investigations specifically devised to assess prophylactic antibiotic strategies, encompassing agent selection, the timing of re-dosing, and the total duration, in patients undergoing prolonged neurosurgical procedures are necessary to resolve this clinically significant issue.

This study has several limitations. The single-center design and relatively small number of infection cases may have limited the statistical power. The single-point sampling intraoperatively failed to capture the dynamic evolution of cytokine levels over time. Other potentially valuable biomarkers, such as procalcitonin (PCT), C-reactive protein (CRP) or emerging microbiological markers, were not assessed. In future studies, researchers could implement longitudinal and dynamic sampling strategies for CSF or serum postoperatively to map cytokine trajectories, as this may offer higher predictive value than static measurements. Researchers could also integrate host inflammatory markers with molecular diagnostics, such as mNGS, to construct more robust diagnostic models. Finally, the exploratory nature of this study suggests that the findings should be interpreted with caution, and that the absence of statistical significance does not exclude a potential underlying biological relationship, which may require validation in larger, multi-institutional cohorts.

## 6. Conclusions

In this exploratory study, single-point intraoperative CSF cytokine concentrations were not significantly associated with PII after stringent correction for multiple testing. These findings indicate that single-point intraoperative cytokine measurement did not yield a validated clinical prediction tool in patients undergoing craniotomy for benign brain tumors in this small exploratory cohort. The unadjusted and direction-corrected findings for MCP-1 and IL-4 remain exploratory and require validation. Future investigations with larger sample sizes and longitudinal sampling designs may be required to further clarify temporal immune dynamics.

## Figures and Tables

**Table 1:** A comparison of baseline characteristics and intraoperative CSF cytokine levels between the non-infection and infection groups.

Variable	Infection Group (*n* = 18)	Non-Infection Group (*n* = 116)	*p*-Value	q-Value
Age (y)	47.78 ± 13.44	54.86 ± 12.49	0.028 *	
Sex, *n* (%)			1	
Male	10 (55.6)	62 (53.4)		
Female	8 (44.4)	54 (46.6)		
Hypertension, *n* (%)	0 (0.0)	26 (22.4)	0.055	
Diabetes, *n* (%)	0 (0.0)	6 (5.2)	0.708	
Tumor type, *n* (%)			1	
Vestibular schwannoma	12 (66.7)	74 (63.8)		
Meningioma	6 (33.3)	42 (36.2)		
Surgical site, *n* (%)			0.938	
Cerebellopontine angle region	13 (72.7)	79 (68.1)		
Parasagittal region	5 (27.8)	37 (31.9)		
Operative duration (min)	390.00 [245.00, 428.00]	244.50 [140.00, 325.00]	0.005 *	
Blood loss (mL)	100.00 [100.00, 200.00]	100.00 [30.00, 100.00]	0.059	
Cytokines (pg/mL)				
MIP-1β	14.24 [3.71, 17.40]	13.11 [4.81, 56.68]	0.381	0.722
MIP-1α	5.87 [5.33, 7.66]	6.85 [4.13, 14.15]	0.676	0.724
MCP-1	57.78 [37.06, 77.40]	116.03 [57.24, 318.67]	0.003 *	0.051
IL-17	4.80 [2.91, 7.06]	5.04 [2.60, 15.20]	0.382	0.649
IL-13	5.13 [4.78, 5.79]	6.01 [3.95, 12.91]	0.54	0.722
IL-12	5.59 [3.67, 7.72]	5.78 [2.50, 16.75]	0.657	0.724
IL-10	8.24 [5.75, 9.32]	9.17 [4.64, 21.86]	0.382	0.649
IL-8	8.95 [7.85, 11.82]	10.52 [7.31, 15.79]	0.21	0.649
IL-6	17.15 [15.53, 25.88]	23.04 [13.30, 46.90]	0.175	0.649
IL-1β	7.17 [5.91, 9.65]	9.93 [3.59, 32.93]	0.341	0.649
IL-1α	3.80 [3.38, 4.19]	4.68 [1.78, 13.60]	0.404	0.649
IFN-γ	23.42 [20.14, 47.00]	40.29 [14.46, 161.52]	0.196	0.649
G-CSF	17.05 [14.88, 17.30]	17.67 [10.55, 48.16]	0.938	0.940
TNF-α	5.35 [5.28, 6.45]	5.70 [4.76, 6.53]	0.629	0.724
IL-4	24.38 [23.52, 29.71]	28.18 [24.73, 33.94]	0.032 *	0.261
IFN-α	2.22 [2.02, 2.67]	2.13 [1.80, 2.39]	0.251	0.649

Age is presented as mean ± SD, and all other continuous variables are presented as median [IQR]. Categorical variables are presented as *n* (%). Q-values represent FDR-adjusted *p*-values for cytokine comparisons. * *p* < 0.05. Abbreviations: CSF, cerebrospinal fluid; IQR, interquartile range; FDR, false discovery rate; MIP, macrophage inflammatory protein; MCP-1, monocyte chemoattractant protein-1; IL, interleukin; IFN, interferon; G-CSF, granulocyte colony-stimulating factor; TNF, tumor necrosis factor.

**Table 2:** Univariate analysis between variables and intracranial infection.

Variable	Unit	OR (95% CI)	*p*-Value	FDR-Corrected *p*-Value
Age	Per 10-year	0.67 (0.46–0.97)	0.033 *	-
Sex	-	0.92 (1.05–1.34)	0.868	-
Tumor type	-	0.88 (0.31–2.52)	0.813	-
Surgical site	-	0.82 (0.27–2.47)	0.726	-
Operative duration	Per 30 min	1.19 (1.05–1.34)	0.005 *	-
Blood loss	Per 100 mL	1.34 (0.97–1.99)	0.075	-
MIP-1β	Per 10 pg/mL	0.89 (0.76–1.04)	0.147	0.194
MIP-1α	Per 10 pg/mL	0.52 (0.20–1.31)	0.165	0.194
MCP-1	Per 10 pg/mL	0.92 (0.86–1.00)	0.043 *	0.172
IL-17	Per 10 pg/mL	0.62 (0.32–1.19)	0.147	0.194
IL-13	Per 10 pg/mL	0.43 (0.14–1.29)	0.131	0.194
IL-12	Per 10 pg/mL	0.71 (0.40–1.25)	0.237	0.263
IL-10	Per 10 pg/mL	0.67 (0.39–1.16)	0.151	0.194
IL-8	Per 10 pg/mL	0.57 (0.26–1.26)	0.165	0.194
IL-6	Per 10 pg/mL	0.80 (0.61–1.05)	0.104	0.194
IL-1β	Per 10 pg/mL	0.80 (0.58–1.10)	0.160	0.194
IL-1α	Per 10 pg/mL	0.54 (0.24–1.22)	0.138	0.194
IFN-γ	Per 10 pg/mL	0.95 (0.90–1.02)	0.145	0.194
G-CSF	Per 10 pg/mL	0.83 (0.66–1.06)	0.136	0.194
TNF-α	Per 1 pg/mL	0.98 (0.77–1.24)	0.865	0.865
IL-4	Per 10 pg/mL	0.36 (0.14–0.96)	0.041 *	0.172
IFN-α	Per 1 pg/mL	1.31 (0.64–2.68)	0.468	0.493

Age is analyzed per 10-year increase, operative duration per 30 min increase, and blood loss per 100 mL increase. Cytokines are analyzed per 10 pg/mL increase except TNF-α and IFN-α (per 1 pg/mL). *p*-values for clinical variables are unadjusted. * *p* < 0.05 indicates statistical significance.

**Table 3:** ROC curve analysis.

	AUC	*p*-Value	95% CI	Optimal Cutoff Point	Sensitivity	Specificity	Youden Index
Age	0.665	0.025	0.526–0.803	53.50	0.778	0.552	0.330
MCP-1	0.716	0.003	0.595–0.838	80.82	0.778	0.672	0.450
IL-4	0.657	0.032	0.540–0.774	26.61	0.667	0.724	0.391
Operative duration	0.708	0.002	0.578–0.838	389.5	0.556	0.862	0.418

## Data Availability

The raw data supporting the conclusions of this article will be made available by the authors on request.

## Supplementary Materials

The following supporting information can be downloaded at: https://www.mdpi.com/article/10.3390/jcm15135119/s1, Table S1: Case-level information of 18 intracranial infection patients.
